# ZNT7 binds to CD40 and influences CD154‐triggered p38 MAPK activity in B lymphocytes—a possible regulatory mechanism for zinc in immune function

**DOI:** 10.1002/2211-5463.12211

**Published:** 2017-03-27

**Authors:** Surapun Tepaamorndech, Pieter Oort, Catherine P. Kirschke, Yimeng Cai, Liping Huang

**Affiliations:** ^1^ Integrative Genetics and Genomics Graduate GroupUniversity of California DavisCAUSA; ^2^ Food Biotechnology Research UnitNational Center for Genetic Engineering and BiotechnologyPathum ThaniThailand; ^3^ Obesity and Metabolism Research UnitUSDA/ARS/Western Human Nutrition Research CenterDavisCAUSA; ^4^ Graduate Group of Nutritional BiologyUniversity of California DavisCAUSA; ^5^ Present address: Astrona BiotechnologiesHM Clause Innovation Center28605 County Road 104DavisCA95618USA

**Keywords:** B lymphocytes, CD154, CD40, p38 MAPK, ZNT7

## Abstract

Zinc deficiency impairs the immune system leading to frequent infections. Although zinc is known to play critical roles in maintaining healthy immune function, the underlying molecular targets are largely unknown. In this study, we demonstrate that zinc is important for the CD154–CD40‐mediated activation of downstream signaling pathways in human B lymphocytes. CD40 is a receptor localized on the cell surface of many immune cells, including B lymphocytes. It binds to CD154, a membrane protein expressed on antigen‐activated T helper (Th) lymphocytes. This CD154‐CD40 interaction leads to B‐cell activation. We showed that cellular zinc deficiency impaired the CD154‐CD40‐mediated p38 mitogen‐activated protein kinase (p38 MAPK) phosphorylation. We also showed that zinc supplemental treatment of B lymphocytes had limited effect on this CD40‐mediated p38 MAPK signaling. Most importantly, we demonstrated that the zinc transporter protein zinc transporter 7 (ZNT7) interacted with CD40 using immunoprecipitation analyses. *ZNT7* knockdown in B lymphocytes had a negative effect on the cell surface expression of CD40. Consequently, the CD40‐mediated p38 MAPK signaling transduction was down‐regulated in *ZNT7* KD B lymphocytes. Conversely, this p38 MAPK signaling activity was up‐regulated by overexpression (OE) of *ZNT7* in B lymphocytes. Moreover, we found that *ZNT7* knockdown in B lymphocytes constitutively up‐ and down‐regulated the inhibitor of i kappa B kinase and AKT serine/threonine kinase phosphorylation, respectively, which implies the activation of survival signaling in *ZNT7* KD B cells. We conclude that CD40 is the target molecule for ZNT7 in regulation of immune function of B lymphocytes.

**Abbreviations**
AKT: AKT serine/threonine kinase
CD154: cluster of differentiation 154
CD30: cluster of differentiation 30
CD40: cluster of differentiation 40
CHO: Chinese hamster ovarian cells
ER: endoplasmic reticulum
ERK: extracellular signal‐regulated kinase
GST: glutathione S‐transferase
IKK: IκB kinase
IκB: inhibitor of i kappa B
JAK3: Janus kinase 3
JNK: c‐Jun N‐terminal kinase
KD: knockdown
KO: knockout
MHC: major histocompatibility complex
MKKK: mitogen‐activated protein kinase kinase kinase
MKK: mitogen‐activated protein kinase kinase
NF‐κB: nuclear factor kappa‐light‐chain enhancer of activated B cells
OE: overexpression
p38 MAPK: p38 mitogen‐activated protein kinase
shRNA: short hairpin RNA
SLC30A: solute carrier 30A
SLC39A: solute carrier 39A
STAT3: signal transducer and activator of transcription 3
TCR: T‐cell receptor
Th1/Th2: T helper cell 1/T helper cell 2
TNFR: tumor necrosis factor receptor
TRAF: TNFR‐associated factor
ZIP: zinc, iron permease
ZNT7: zinc transporter 7

The B lymphocyte functions in the humoral immunity in humans 1. B‐cell activation is induced by binding to antigens, and the activation can be both T cell‐dependent and ‐independent 2. In the T cell‐dependent pathway, B cells bind to antigens which are presented by antigen‐presenting cells via antigen‐binding receptors. The antigen‐bound receptor is then internalized through receptor‐mediated endocytosis 3. The internalized antigen is processed and then coupled with major histocompatibility complex (MHC)‐II molecules on the cell membrane. Subsequently, T helper (Th) cells bind to the antigen‐presenting complex via the T‐cell receptor (TCR)‐MHC‐II‐peptide‐binding mechanism 4. Activated Th cells express a membrane‐bound protein of the tumor necrosis factor receptors (TNFRs), cluster of differentiation 154 (CD154) 5. It is a ligand of cluster of differentiation 40 (CD40), another member of TNFRs expressed abundantly on the surface of the B‐cell. Interaction of CD154 with CD40 results in the recruitment of Janus kinase 3 (JAK3) 6 and/or several TNFR‐associated factor adaptor proteins, such as TRAF1 7, TRAF2 7, TRAF3 8, TRAF5 9, and TRAF6 10 as well as TNFR‐associated factor (TRAF)‐interacting proteins to form a signalosome 11. These proteins serve as mediators of signal transduction pathways including p38 mitogen‐activated protein kinase (p38 MAPK) 12, c‐Jun N‐terminal kinase (JNKs) 13, nuclear factor kappa‐light‐chain‐enhancer of activated B cells (NF‐κB) 10, AKT serine/threonine kinase (AKT) 14, and signal transducer and activator of transcription 3 (STAT3) 6, which triggers intracellular signaling cascades leading to B‐cell proliferation, survival, immunoglobulin isotype switching, and somatic hypermutation 15.

Zinc is an essential trace metal for body growth 16. In humans, zinc deficiency causes impaired immune function leading to increased risk of infections, such as upper respiratory infection and diarrhea 17, 18. It has also been shown that marginal zinc deficiency negatively affects both adaptive immunity and humoral immunity 19. In the thymus, abnormal cellular zinc homeostasis decreases T‐cell maturation and activation with altered Th cell 1/T helper cell 2 (Th1/Th2) responses. Zinc deficiency also impairs B‐cell development and differentiation in response to immune stimuli, such as vaccination. Cellular zinc homeostasis is tightly regulated by two families of zinc transporters, solute carrier 30A (SLC30A; ZNT) and solute carrier 39A [SLC39A; zinc, iron permease (ZIP)] in humans. The ZNT and ZIP families contain 10 and 14 members, respectively. ZNT proteins mainly function to reduce cytoplasmic zinc levels by removing cytoplasmic zinc out of the cell, or shifting it into organelles when zinc is replete 20 while ZIP proteins do the opposite 21.

During a search for a potential protein–protein interaction partner for zinc transporter 7 (ZNT7), we came across the CD40 protein 22. CD40 has been shown to interact with many protein partners to transduce signals from activated T cells to B cells or other immune cells, such as macrophages, via the CD154‐CD40 signaling pathway. In a large‐scale mapping of human protein–protein interactions by mass spectrometry, Ewing *et al*. 22 showed that CD40 could partner with two zinc transporters, ZNT7 23 and ZIP7 24, 25, 26. Both ZNT7 and ZIP7 are localized on the membrane of the Golgi/endoplasmic reticulum (ER) and vesicular compartments. They function to transport zinc in opposite directions 23, 24. mRNA expression profiles of *ZNT7* and *ZIP7* are similar to each other: both are widely expressed but more abundantly in tissues containing high zinc levels 23, 24, 26, 27, 28. The possible interaction of CD40 with ZNT7 or ZIP7 suggests that zinc may be an important regulator or cofactor for the CD40‐mediated signal transduction in immune cells.

Overexpression (OE) of *ZnT7* in Chinese hamster ovarian cells (CHO) exposed to high zinc results in accumulation of zinc in the Golgi apparatus and vesicles 23. Mice with a null‐mutation of *Znt7* are marginally zinc deficient with serum zinc concentrations ~ 20% lower than the wild‐type (wt) control 29. Embryonic fibroblasts isolated from *Znt7* knockout (KO) mice contain only ~ 50% of cellular zinc compared to the wt littermates 29. *Znt7* KO mice gain less weight than the wt control due to a defect in fat accumulation in adipocytes 30. The low body weight in *Znt7* KO mice cannot be corrected by feeding these KO mice with a zinc supplemental diet (180 mg zinc·kg^−1^ diet) 29. Furthermore, although *Znt7* KO mice are lean, they are susceptible to diet‐induced insulin resistance in muscle and fat tissues 30, 31.

Our recent studies suggest that *Znt7* KO, or siRNA silencing *Znt7* expression negatively affects cellular signaling pathways, including insulin/insulin receptor‐mediated AKT activity 30, 31. In addition, we found that the circulating B and T lymphocytes in *Znt7* KO mice were significantly reduced compared to the wt littermate control (C. P. Kirschke & L. Huang, unpublished data). Given that CD40 potentially interacted with ZNT7 (which was uncovered in a bait–prey pair mapping of proteins of significant biomedical interest) and *Znt7* KO mice had low B and T lymphocyte numbers in the circulation, we hypothesized that ZNT7 might play a critical role in the CD40‐mediated signaling transduction in B lymphocytes.

Here, we report, for the first time, the molecular mechanism of how zinc affects immune function. We demonstrated that the activation of the CD40 ligand (CD154)‐induced p38 MAPK was negatively affected by cellular zinc deficiency in Raji B lymphocytes while zinc supplementation had little influence on the activity of p38 MAPK when cellular zinc was replete. We also confirmed CD40–ZNT7 interaction by immunoprecipitation analysis with either glutathione S‐transferase‐tagged ZNT7 or endogenous ZNT7 isolated from Raji B lymphocytes. Lastly, we showed that alternation of *ZNT7* expression in Raji B cells was associated with the cell surface expression level of CD40 and the downstream activity of the CD154‐induced signaling pathways.

## Results

### Zinc affects the CD154‐induced activation of p38 MAPK in Raji B lymphocytes

**Figure 1:**
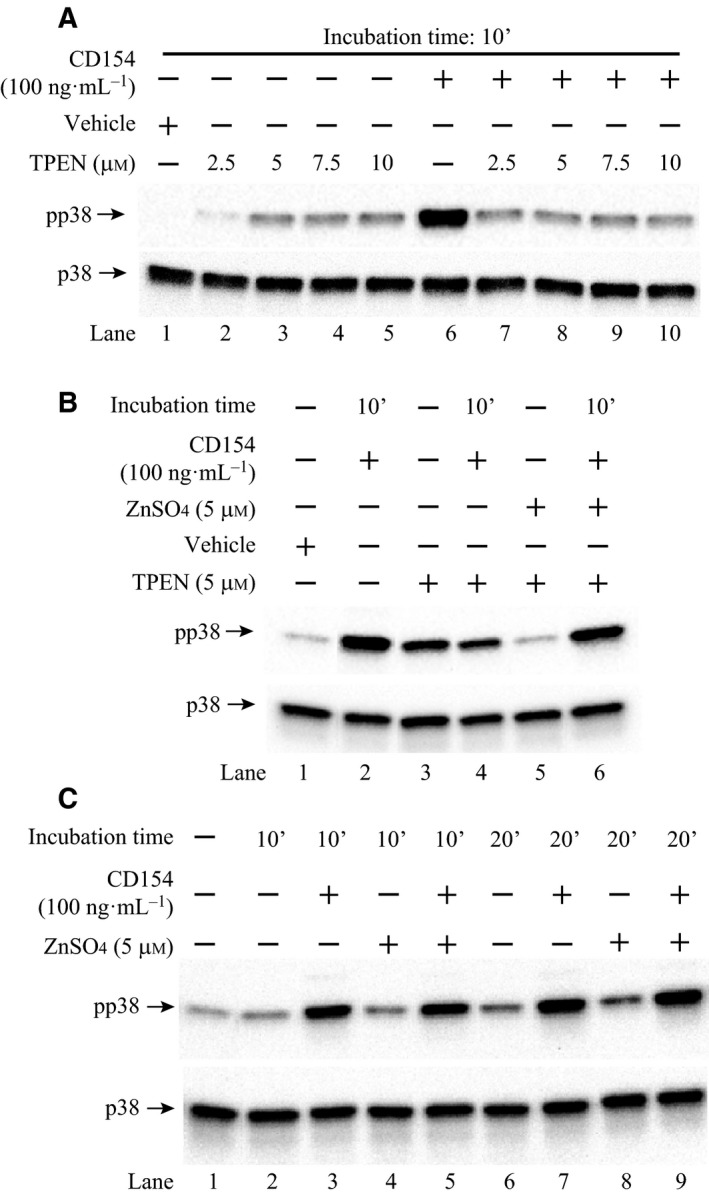
The effect of changes in cellular zinc concentrations on the p38 MAPK activity in Raji B lymphocytes. (A) Depletion of cellular zinc attenuated the p38 MAPK activity stimulated by CD154 in Raji B cells. Raji B cells were seeded into 6‐well plates at 3 × 10^6^ in serum‐free RPMI 1640 media with TPEN at the indicated concentrations or vehicle at 37 °C for 2 h. Cells were then stimulated with CD154 at 37 °C for 10 min and lysed for western blot analysis. (B) TPEN‐induced reduction in the p38 MAPK activity was restored by zinc treatment in Raji B cells. Raji B cells were seeded into six‐well plates at 3 × 10^6^ in serum‐free RPMI 1640 media with or without TPEN (5 μm). As indicated, ZnSO_4_ was added into the medium at 5 μm to negate the TPEN function. The cells were incubated at 37 °C for 2 h. Cells were then stimulated with CD154 at 37 °C for 10 min and lysed for western blot analysis. (C) Zinc treatment increased the CD154‐stimulated p38 MAPK activity in Raji B cells. Raji B cells were seeded into six‐well plates at 3 × 10^6^ in serum‐free RPMI 1640 media with or without ZnSO_4_ at 5 μm. The cells were incubated at 37 °C for 2 h. Cells were then stimulated with CD154 at 37 °C for 10 or 20 min and lysed for western blot analysis. The phosphorylated p38 MAPK was detected by an anti‐phosphor‐p38 MAPK (Thr180/Tyr182) antibody and the total p38 MAPK was detected by an anti‐p38 MAPK antibody.

Recently, a large‐scale mapping study of human protein–protein interaction by mass spectrometry 22 has suggested that ZNT7 may physically interact with CD40. Before we verified this putative interaction between ZNT7 and CD40, we first examined whether changes in cellular zinc concentrations would affect the activity of p38 MAPK, one of the major kinases that is stimulated by the interaction of CD154‐CD40 in Raji B cells. We chose the Raji B cell line because the *CD40* mRNA expression in Raji B cells were highest among B lymphocyte cell lines according to the *CD40* RNA expression pattern revealed by BioGPS (http://biogps.org). We found that stimulation of Raji B lymphocytes with a soluble CD154 (100 ng·mL^−1^) for 10 min activated p38 MAPK (Fig. 1A, comparing lanes 1 & 6). We also found that treatment of Raji B cells with TPEN, a zinc chelator, at 2.5 μm for 2 h before CD154 stimulation inhibited the ligand‐triggered p38 MAPK activation (Fig. 1A, comparing lanes 6 & 7). When TPEN concentrations were increased to 5, 7.5, and 10 μm, no further inhibition of the CD154‐induced p38 MAPK activation was observed (Fig. 1A, comparing lanes 6–10). The total p38 MAPK expression levels were not significantly influenced by the zinc chelation before or after CD154 stimulation (Fig. 1A, comparing lanes 1–5 to 6–10). In addition, we noticed that TPEN treatment at 2.5 μm slightly increased the basal p38 MAPK phosphorylation in Raji B cells (Fig. 1A, comparing lanes 1 & 2), with a more profound augment at 5.0 μm (lane 3). TPEN concentrations at 7.5 and 10 μm did not further increase the basal p38 MAPK activity in Raji B cells (Fig. 1A, comparing lanes 4 & 5 to lane 3).

As we expected, addition of zinc (ZnSO_4_) at 5 μm neutralized the detrimental effect of TPEN on the activation of p38 MAPK after CD154 stimulation in Raji B cells (Fig. 1B, comparing lanes 4 & 6). Zinc treatment also suppressed the TPEN‐induced basal p38 MAPK activity in Raji B cells (Fig. 1B, comparing lanes 3 & 5). Nevertheless, treatment of Raji B cells with ZnSO_4_ (5 μm) alone had little beneficial effects on both basal and CD154‐stimulated p38 MAPK phosphorylation when zinc was replete in Raji B cells (Fig. 1C). Taken together, our results suggest that zinc deficiency has a negative impact on the CD154‐CD40‐mediated p38 MAPK signaling transduction pathway in Raji B cells, whereas zinc supplementation has limited influence on this signaling pathway.

### ZNT7 interacts with CD40

**Figure 2:**
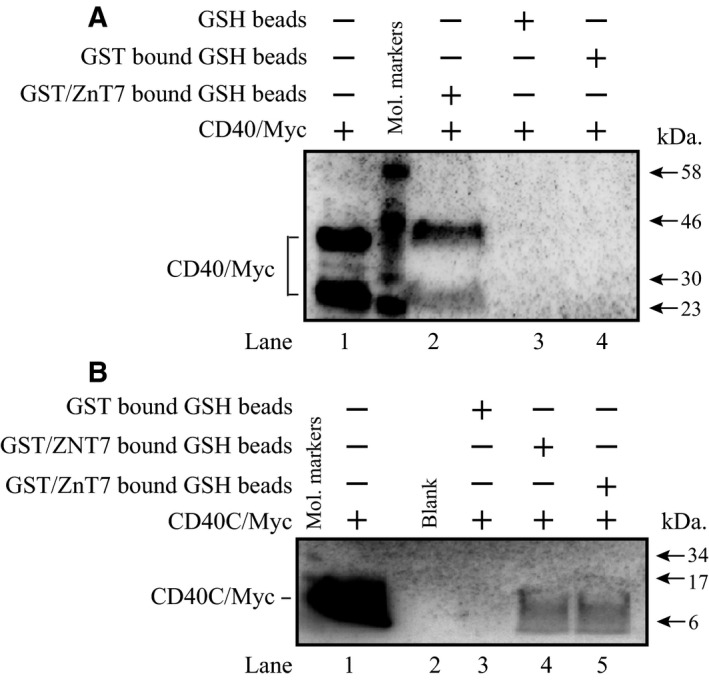
Interaction of ZNT7 with CD40 *in vitro*. Purification of bacteria‐expressed fusion proteins, including mouse GST/ZnT7, human GST/ZNT7, human CD40/Myc, and human CD40C/Myc was described in Materials and methods. (A) Binding of CD40 to mouse ZnT7. Lane 1, the purified CD40/Myc protein. Two protein bands (~ 42 and ~ 25 kDa) were detected. The upper band is the intact CD40 protein and the lower band is a proteolytic product. Lane 2, the pulled‐down CD40/Myc by GST/ZnT7. Lane 3, negative control (CD40/Myc was incubated with GSH beads). Lane 4, negative control (CD40/Myc was incubated with GST‐bound GSH beads). GST/ZnT7, but not GSH or GST‐bound GSH beads, was able to bind to CD40/Myc. (B) Binding of the C‐terminal end of CD40 to either mouse or human ZNT7. Lane 1, the purified CD40C/Myc protein (the C‐terminal 62 amino acids of CD40; ~ 8 kDa). Lane 2, blank. Lane 3, negative control (CD40C/Myc was incubated with GST‐bound GSH beads). Lane 4, the pulled‐down CD40C/Myc by human GST/ZNT7. Lane 5, the pulled‐down CD40C/Myc by mouse GST/ZnT7. The mouse or human GST‐ZNT7 fusion protein, but not the GST‐bound GSH beads, was able to bind to CD40C/Myc. Molecular markers are indicated by arrows. CD40/Myc or CD40C/Myc was detected using an antibody against the Myc tag.

To confirm the interaction of ZNT7 and CD40, which was revealed by the bait–prey pair mapping 22, we constructed and purified glutathione S‐transferase (GST)/ZnT7 (GST‐tagged mouse ZnT7) and CD40/Myc fusion proteins from bacteria and investigated whether ZnT7 could pull‐down CD40 in an *in vitro* pull‐down assay. As shown in Fig. 2A, GST/ZnT7 was able to pull‐down the CD40/Myc fusion protein (lane 2). However, glutathione (GSH) beads and GST‐bound GSH beads could not bind to the CD40/Myc fusion protein (lanes 3 & 4, respectively). We detected two forms of the CD40/Myc fusion protein on the western blot (Fig. 2A, lane 1). One was in the expected size (~ 42 kDa) 32 and the other was smaller (~ 25 kDa). Since the Myc tag was attached to the C‐terminal end of CD40, the smaller band is likely a proteolytic product of the C‐terminal end of CD40. This cleaved peptide is similar to the one (~ 27 kDa) reported in mammalian cells, such as B lymphocytes, macrophages, and dendritic cells 33. Based on the peptide size, the cleavage site could be located within the exodomain of CD40.

The CD40 protein is comprised of one transmembrane domain and the C‐terminal domain, which contains 62 amino acids, faces the cytoplasm of the cell 32. We expressed and purified the Myc‐tagged C‐terminal end of CD40 (CD40C/Myc) and determined whether GST/ZNT7 (human ZNT7) or GST/ZnT7 (mouse ZnT7) could interact with the C‐terminal part of CD40 in an *in vitro* pull‐down assay. As shown in Fig. 2B, both human and mouse ZnT7 proteins were able to bind to the C‐terminal part of CD40, indicating the binding of CD40 to ZNT7/ZnT7 via its C‐terminal end of the protein.

**Figure 3:**
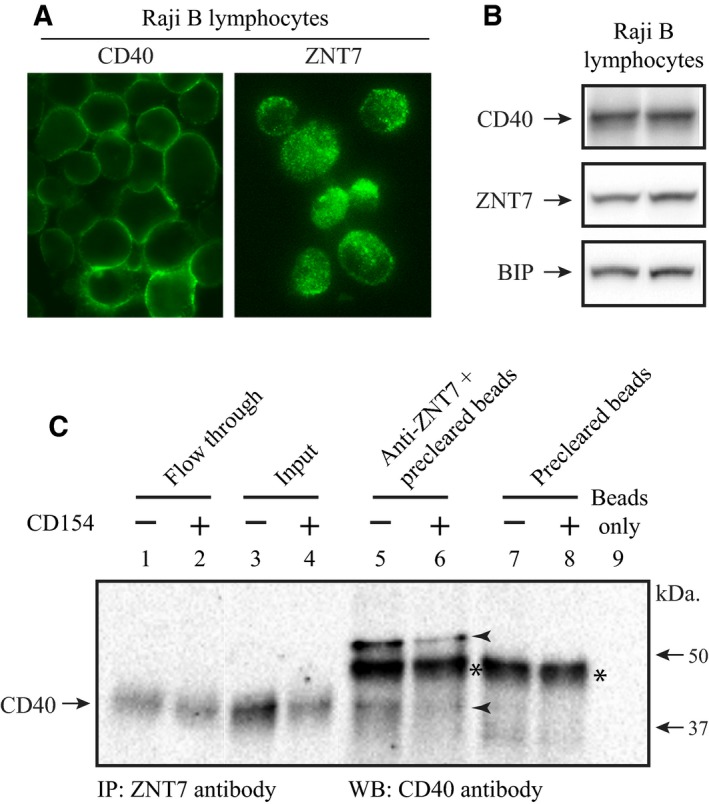
Interaction of ZNT7 with CD40 in Raji B lymphocytes. (A) Subcellular localization of endogenous ZNT7 and CD40 in Raji B cells. Cells were seeded in the complete RPMI 1640 medium without FBS for 2 h before staining. ZNT7 and CD40 were detected by antibodies against ZnT7 23 and CD40, respectively. (B) Expression of endogenous ZNT7 and CD40. Proteins were detected using anti‐ZnT7 and anti‐CD40 antibodies by western Blot analysis. The expression of BIP was used as the loading control. The two lanes are the protein lysate harvested from two independent experiments. (C) Immunoprecipitation. Raji B cells treated with or without CD154 (100 ng·mL^−1^) were harvested for the immunoprecipitation assay as described in Materials and methods. CD40 was immunoprecipitated by the antibody against human ZNT7 and detected by the antibody against CD40 in western blot analysis. Interaction of ZNT7 with CD40 was observed in lanes 5 and 6 (arrowheads). Two CD40 protein bands were pulled down by the ZNT7 antibody, ~ 40 and ~ 60 kDa. The lower band is the intact CD40 protein and the upper band may represent an additional protein bound to the ZNT7‐CD40 complex. The asterisk represents a nonspecific binding of protein to the goat anti‐(rabbit IgG) magnetic beads. IP, immunoprecipitation; WB, western blot assay. Molecular markers are indicated by arrows. The amounts of the input (lanes 3 & 4) were 1.6% of the starting lysate used in the IP assay.

We next sought to determine whether endogenous ZNT7 could interact with CD40. We first showed that ZNT7 and CD40 proteins were abundantly expressed in Raji B lymphocytes by immunofluorescence microscopy (Fig. 3A) and western blot analysis (Fig. 3B). CD40 was detected on the cell surface of Raji B cells (no permeabilization) with an anti‐CD40 antibody against the N‐terminal end of the protein while ZNT7 was localized intracellularly with a punctate staining pattern (Fig. 3A). It is worth noting that some ZNT7‐positively stained fine punctates were localized along the plasma membrane (Fig. 3A). Next, we confirmed the interaction of the endogenous ZNT7 with CD40 in Raji B cells by cross‐linking immunoprecipitation using a ZnT7 antibody to pull‐down CD40. Western blot assay using a CD40 antibody displayed two protein bands with the sizes in ~ 40 kDa and ~ 60 kDa (Fig. 3C, lanes 5 & 6). The upper protein band was more abundant than the lower protein band. The lower protein band is in the expected size for CD40 and the upper protein band could be the result of an additional protein(s) bound to the ZNT7–CD40 complex in the cell. Notably, we showed that ZNT7 was able to interact with CD40 in both CD40 ligand‐stimulated and unstimulated conditions. Taken together, our data demonstrated that ZNT7 could interact with CD40 at both the basal and CD154‐stimulated conditions in Raji B cells.

### Reduction in ZNT7 expression decreased cell surface expression of CD40 in Raji B lymphocytes

**Figure 4:**
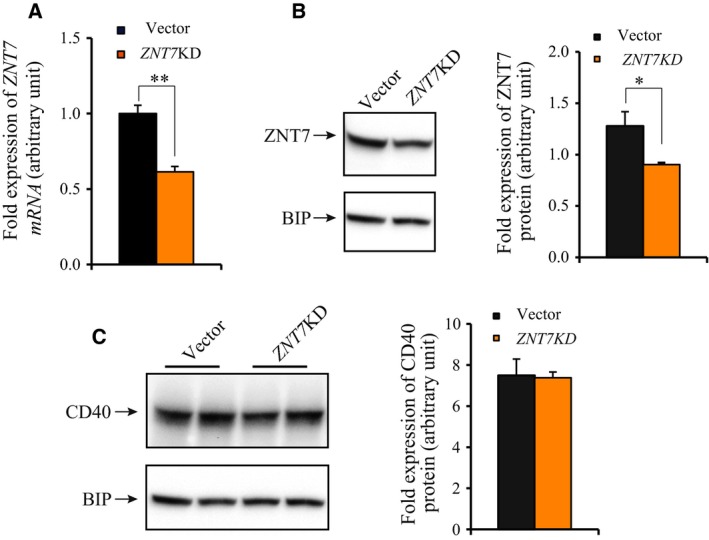
Analysis of *ZNT7* KD Raji B cell line. (A) mRNA expression of *ZNT7* in *ZNT7* KD Raji B cells. *ZNT7* mRNA expression in *ZNT7* KD and vector control Raji B cell lines was measured using a SYBR‐based quantitative RT‐PCR. *ACTB* was used as the internal control. Data were obtained from three independent experiments. Relative expression of *ZNT7* mRNA in *ZNT7* KD Raji B cells to the control was plotted. (B) ZNT7 protein expression in *ZNT7* KD Raji B cells. ZNT7 was detected using an anti‐ZnT7 antibody 23. The expression of BIP was used as the loading control. (C) CD40 protein expression in *ZNT7* KD Raji B cells. CD40 was detected using an anti‐CD40 antibody purchased from Santa Cruz Biotechnology. The expression of BIP was used as the loading control. Data were presented as mean ± SE (*n* = 3) of three independent experiments. Representative western blots are presented. *P < 0.05; **P < 0.01.

To understand ZNT7's role in the CD154‐CD40 complex‐induced signal transduction pathways in Raji B cells, we established a stable *ZNT7* knockdown (*ZNT7*KD) Raji B cell line using a lentiviral short hairpin RNA (shRNA) against the human ZNT7 (Sigma‐Aldrich, St. Louis, MO, USA). We confirmed that the mRNA expression of *ZNT7* in *ZNT7*KD Raji cells was reduced to ~ 60% of the normal level by quantitative RT‐PCR (Fig. 4A). Most importantly, we showed that ZNT7 protein expression in *ZNT7*KD Raji B cells was reduced to ~ 70% of the level in the control cells (Fig. 4B). Next, we investigated whether reduced *ZNT7* expression in Raji B cells affected CD40 expression at both mRNA and protein levels using quantitative RT‐PCR and western blot assays. We found that *ZNT7*KD in Raji B cells had little to no effect on both CD40 mRNA expression (data not shown) and protein expression (Fig. 4C).

**Figure 5:**
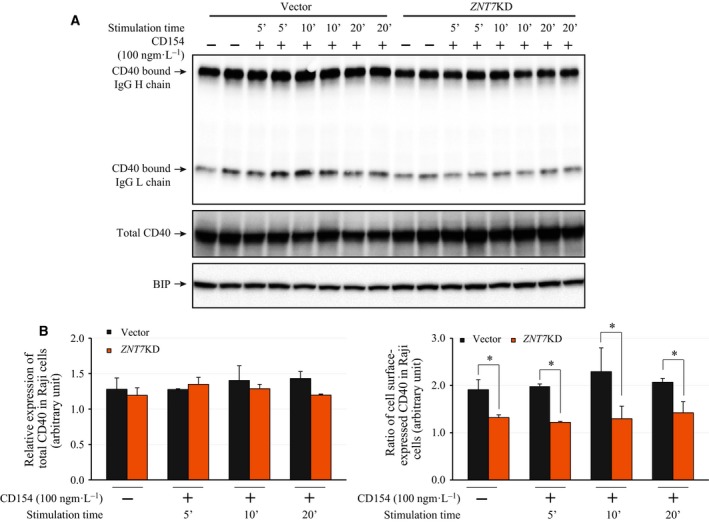
Cell surface expression of CD40 in *ZNT7* KD and control Raji B lymphocytes. (A) Western analysis of total CD40 protein levels and cell surface‐expressed CD40 levels (indicated by the levels of the CD40‐bound IgG chains) in *ZNT7* KD and control Raji B lymphocytes. BIP was used as a control. (B) Densitometry analysis of CD40 expression on the western blots. The amount of expressed BIP was used for normalization. (C) Ratio of cell surface‐expressed CD40. The densitometry of CD40‐bound IgG H and L chains was determined and normalized to the total expressed CD40. Data are mean ± SD from two independent experiments and a representative western blot is shown. *P < 0.05.

CD40 on the cell surface is constitutively internalized, through which the amount of CD40 on the cell surface is regulated. The concentration of CD40 on the cell surface available for CD154 binding determines the degree of activation of downstream signaling transduction pathways 34. As such we examined the surface expression of CD40 in *ZNT7*KD Raji B cells before and after CD154 stimulation. Fixed *ZNT7*KD and vector control cells were probed with a mouse monoclonal antibody raised against the extracellular domain of CD40. The cell surface expression of CD40 was indirectly determined by the amount of antibodies bound to the cell surface. As shown in Fig. 5, *ZNT7*KD in Raji B cells resulted in about 30–50% reduction in the expression of CD40 on the cell surface in both basal and stimulated conditions. It is worth noting that CD154 stimulation up to 20 min did not have negative effects on the cell surface expression of CD40 in both *ZNT7*KD and control Raji B cells. Taken together, our data suggest that ZNT7 plays a critical role in regulation of cell surface expression of CD40 in Raji B cells.

### Reduction in ZNT7 expression negatively affects CD154‐CD40‐stimulated signal transduction pathways in Raji B lymphocytes

**Figure 6:**
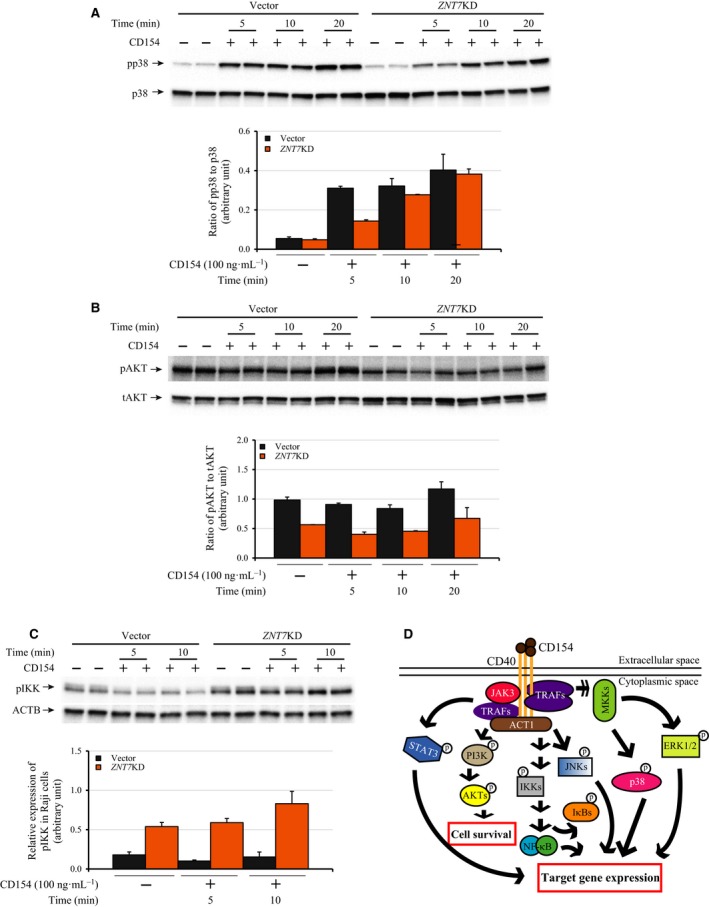
Effect of *ZNT7* knockdown on the CD40 signaling pathways in Raji B lymphocytes. Z*NT* *7* KD and control Raji B cells were seeded and cultured for 48 h. CD154 stimulation (100 ng·mL^−1^) was carried out at 0, 5, 10, or 20 min at 37 °C in duplicate. After stimulation, the cells were washed and lysed for western blot analysis. (A) Phosphorylation of p38 MAPK. (B) Phosphorylation of AKTs. Total p38 MAPK and AKTs were also examined. (C) Phosphorylation of IKKs. ACTB was used for the loading control. The activations of STAT3 and ERK1/2 signaling pathways were not detectable in both *ZNT7* KD and control Raji B cells after CD154 stimulation (data not shown). Experiments were each performed twice and representative results are shown. (D) The CD154‐CD40 signaling pathways in B lymphocytes. CD154‐stimulated CD40 signaling activates multiple downstream signaling pathways in B lymphocytes that influence B lymphocyte proliferation, isotype switching, differentiation, and survival. The diagram represents the pathways that were examined in this study. Single arrow, direct stimulation; multiple arrows, multistep simulation; p with a circle, activated by phosphorylation.

We next sought to investigate whether decreased cell surface expression of CD40 in *ZNT7*KD Raji B cells had a significant impact on the CD154‐CD40‐induced activities of downstream signal transduction pathways. It has been reported that CD154–CD40 interaction recruits diverse effector components, which can trigger various downstream signal transduction pathways, including p38 MAPK, ERK1/2 MAPK, JNKs, inhibitor of i kappa B (IκB) kinase (IKK), AKTs, and STAT3 (Fig. 6D). We examined whether *ZNT7*KD altered these signaling transduction pathways after CD154 stimulation in Raji B cells. As shown in Fig. 6A, activation of the p38 MAPK signal pathway could be detected at 5 min after CD154 (100 ng·mL^−1^) stimulation in *ZNT7*KD and control Raji B cells. Importantly, we observed that the phosphorylation of p38 MAPK and AKTs was lower in *ZNT7*KD Raji B cells than the control (Fig. 6A,B). The ratio of phosphor‐p38 MAPK/total p38 MAPK and phosphor‐AKTs/total AKTs were decreased in *ZNT7*KD Raji B cells compared with the control (Fig. 6A,B). Interestingly, we observed that CD154–CD40 interaction slightly down‐regulated the activity of IKKs (the IKKs that phosphorylates IκB α resulting in the activation of NF‐κB) in the control but not in the *ZNT7*KD Raji B cells (Fig. 6C). Nevertheless, we did not detect any significant differences in the activation of the JNKs signaling pathway between *ZNT7*KD and the control after CD154 stimulation (data not shown). Neither did we observe any detectable phosphorylation of ERK1/2 MAPK and STAT3 in both *ZNT7*KD and control Raji cells after CD154 stimulation up to 20 min (data not shown). Together, our results suggest that down‐regulation of the cell surface expression of CD40 in *ZNT7*KD Raji B cells impairs the CD154‐CD40‐induced p38 MAPK and AKT signal transduction pathways.

### Overexpression of ZNT7 stimulates CD154‐induced activations of p38 MAPK and AKTs and cell surface expression of CD40

**Figure 7:**
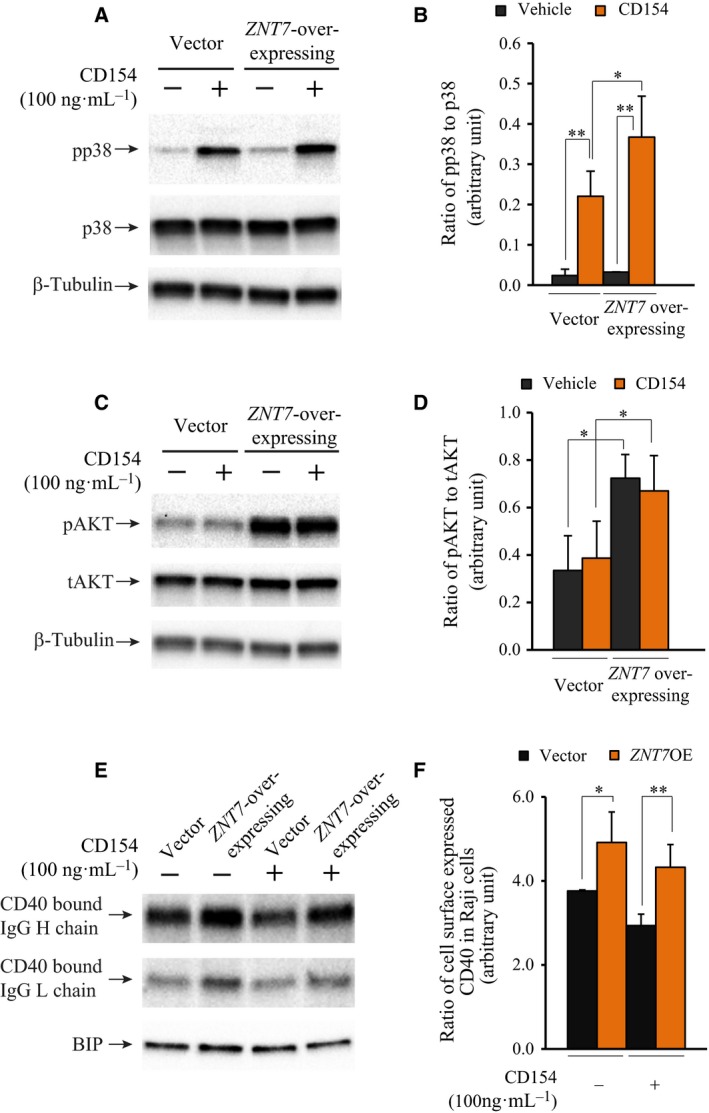
Effect of *ZNT7* OE on the activations of p38 MAPK and AKTs and cell surface expression of CD40 in Raji B cells. Raji B cells overexpressing *ZNT7* were stimulated with CD154 (100 ng·mL^−1^) at 37 °C for 5–10 min before harvesting. Western blot analysis was performed as described in Materials and methods. The expression of β‐Tubulin or BIP was used as the loading control. (A) Activation of p38 MAPK in *ZNT7*‐overexpressing Raji B cells. (B) Ratio of phosphor‐p38 MAPK to the total p38 MAPK. (C) Activation of AKTs in *ZNT7*‐overexpressing Raji B cells. (D) Ratio of phosphor‐AKTs to the total AKTs. (E) Cell surface expression of CD40 (indicated by the levels of the CD40‐bound IgG chains). (F) Densitometry analysis of the CD40‐bound IgG heavy chain. The amount of expressed BIP was used for the normalization. Experiments were each performed twice with duplication and representative western blots are shown. *P < 0.05; **P < 0.01.

We have demonstrated that reduction in *ZNT7* expression by a *ZNT7* gene‐silencing approach impaired the activation of p38 MAPK after CD154 stimulation as well as the basal activity of AKTs in Raji B cells (Fig. 6). To confirm the role of ZNT7 in the CD154‐CD40‐mediated signal transduction pathways in Raji B cells, we generated a *ZNT7*‐overexpressing Raji B cell line using a mammalian‐expressing plasmid that carried a full‐length cDNA of *ZNT7*. We selected three individual cell clones for the *ZNT7* line with the *ZNT7* mRNA expression level at ~ 50% higher than the vector control (two lines, data not shown). We next examined the activities of p38 MAPK and AKTs in these cell lines treated with or without CD154. As shown in Fig. 7A,B, OE of *ZNT7* increased the activation of p38 MAPK by 70% compared to the control after CD154 stimulation for 10 min. We also observed that *ZNT7* OE in Raji B cells strongly enhanced the phosphorylation of AKTs in the basal condition (Fig. 7C,D), consistent with the results obtained from the *ZNT7*KD Raji B cell line (Fig. 6B). Interestingly, CD154 stimulation seemed to have little to no impact on the AKT phosphorylation in either *ZNT7*‐overexpressing or vector control Raji B cells (Fig 7C,D), again consistent with the results obtained from *ZNT7*KD Raji B cells (Fig. 6B). In addition, we examined the cell surface expression of CD40 in *ZNT7*‐overexpressing Raji B cells and compared it to the control. Again the cell surface expression of CD40 was indirectly determined by the amount of antibodies bound to the cell surface using a biotin‐conjugated antibody against human CD40. As shown in Fig. 7E,F, *ZNT7* OE in Raji B cells lead to about 30–47% increase in the expression of CD40 on the cell surface in both basal and stimulated conditions, consistent with the results obtained from *ZNT7*KD Raji B cells (Fig. 5). Taken together, our data suggest that ZNT7 affects levels of CD40 expression on the cell surface, and ZNT7 is involved in the CD154‐CD40‐mediated p38 MAPK activation in Raji B lymphocytes.

## Discussion

It is known that individuals with moderate or severe zinc deficiency are prone to infections due to impaired immune function 19. However, the molecular targets and mechanisms underlying the action of zinc in the immune system are still poorly understood. Here, we report a role of ZNT7 in CD154‐triggered CD40 signal transduction pathways in human Raji B lymphocytes. We demonstrated that cellular zinc deficiency induced by TPEN could inhibit CD154‐stimulated p38 MAPK phosphorylation in Raji B lymphocytes. We also demonstrated that this TPEN‐induced inhibition of p38 MAPK activation in Raji B cells could be reversed by the addition of zinc to the cell. In addition, our study revealed that when Raji B cells were zinc replete, the addition of supplemental zinc to the cell did not further augment the CD40‐mediated activation of p38 MAPK in the cell. These results suggest that, in humans, prevention of zinc deficiency is the key in maintaining optimal immune system. While zinc supplementation can boost immune function in individuals with zinc deficiency, the beneficial effect of zinc supplementation on immune function (in the aspect of T cell‐mediated B‐cell activation) seems little when cellular zinc is replete.

Cluster of differentiation 40 is a cell surface receptor with its N‐terminal end located in the extracellular space for ligand binding and the C‐terminal end located in the cytosol to be bound by the effector molecules recruited during CD154–CD40 interaction. The effector molecules are organized in both temporal and spatial ways to transduce signals to downstream kinases leading to B‐cell proliferation, survival and antibody isotype switching. In the present study, we demonstrated that ZNT7 was able to physically bind to CD40. We showed that the binding of ZNT7 to CD40 was likely via the cytosolic C‐terminal 62 amino acid tail of the CD40 protein, a domain that many effector molecules bind to during B‐cell activation. We also showed that the interaction of ZNT7 with CD40 was critical for the activations of p38 MAPK, AKTs, and IKK in Raji B cells as these kinase activations were affected by either *ZNT7*KD or OE in Raji B cells. Moreover, we provided evidence indicating that regulation of cell surface expression of CD40 was likely the molecular mechanism underlying the altered activations of CD154‐CD40‐mediated downstream signaling transductions observed in *ZNT7*KD or *ZNT7*OE Raji B lymphocytes.

**Figure 8:**
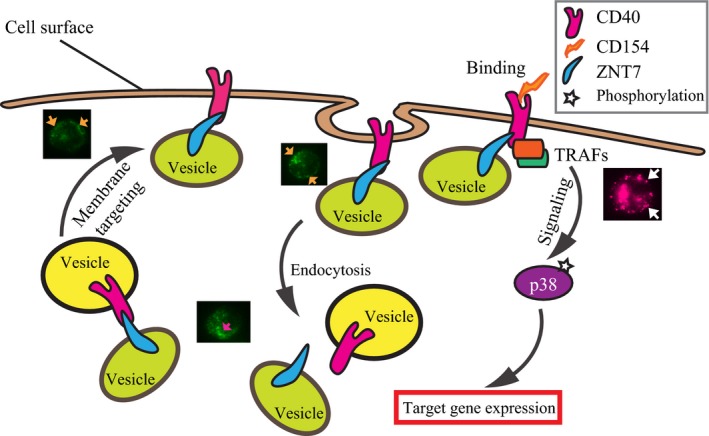
An illustration of the working hypothesis of the regulatory role of ZNT7 in the CD40‐mediated signal transduction. In the B lymphocyte, ZNT7 binds to CD40 that may promote the CD40‐containing vesicles to the cytoplasmic membrane or inhibit CD40 recycling from the cell surface leading to an increase in CD40 expression on the cell surface. As such, ZNT7 indirectly influences the CD40‐mediated downstream signal transduction. This theory is supported by the association of cell surface expression of CD40 with *ZNT7* expression in both *ZNT7* KD and *ZNT7* OE Raji B cells and by the subcellular localization pattern of ZNT7 (shown in green) in Raji B lymphocytes. The arrows in orange and magenta indicate the localization of ZNT7‐containing vesicles along the cytoplasmic membrane and in the cytosol, respectively. On the other hand, ZNT7 may regulate local zinc levels in the CD154‐CD40‐TRAFs complex leading to changes in signal transduction activities. This theory is supported by the effect of ZNT7 expression levels on the CD154‐triggered p38 MAPK activation in Raji B cells and by the *in situ* Zinquin staining of zinc ions (shown in magenta) in ZnT7‐overexpressing CHO cells (shown in maenta) 23. The arrows in white indicate the location of zinc‐rich vesicles along the cytoplasmic membrane.

We speculate two possible mechanisms by which ZNT7 regulates the CD40 signaling transduction in B lymphocytes (Fig. 8). First, ZNT7 may regulate the cellular CD40 trafficking by binding to CD40, to influence either CD40 transport from the vesicular compartment to the cytoplasmic membrane, or CD40 recycling from the cell surface to the cytoplasm. Second, ZNT7 may regulate local zinc concentrations in the CD40‐TRAFs‐kinase signalosome after CD154–CD40 interaction, as TRAFs contain multiple zinc‐binding sites, including RING fingers and zinc fingers 35. ZNT7 may be involved in delivering zinc to these domains to facilitate structural transformation and/or activity of TRAFs after being recruited to the CD40 molecule.

It is interesting to note that ZNT7 mainly affects phosphorylation of p38 MAPK with a limited effect on the activation of extracellular signal‐regulated kinases (ERKs) and JNKs in Raji B cells after CD154 stimulation. MAPKs are the terminal kinases regulated by the upstream kinases, mitogen‐activated protein kinase kinases (MKKs), which themselves are regulated by mitogen‐activated protein kinase kinase kinases (MKKKs). There are at least 20 MKKKs which receive extracellular stimuli and transduce signals downstream to MKKs and MAPKs to regulate cellular response to each stimulus. The specificity of the terminal MAPK activations is largely achieved by the upstream components of MKKKs and MKKs. Given that the upstream kinases that phosphorylate p38 MAPK are MKK3/6, which themselves are regulated by MEKK3 and MEKK4 (the two MKKK members that bind to TRAF6/7), we speculate that ZNT7 plays a critical role in regulation of the activity of the newly formed CD154‐CD40‐TRAF6/7‐MEKK3/4 complex after CD154 stimulation through which a degree of specificity in the signaling cascade is achieved in the B lymphocyte.

The role of zinc as a second messenger in cellular signaling has been demonstrated previously 36. A rise in cytoplasmic‐free zinc concentrations, the so called ‘zinc wave’ after immune cells are activated by external stimuli, is associated with the inhibition of protein phosphatases 36, 37. This zinc‐mediated inhibition of phosphatases increases levels of activated kinases in immune cells during early phases, usually within minutes, of cellular responses to extracellular stimuli 36, 37, 38. It is worth mentioning that SLC39A7 (ZIP7), a zinc transporter 24, 26 with a similar tissue expression pattern and cellular localization to ZNT7, has been shown to be involved in this zinc wave response by delivery of stored zinc into the cytoplasm from either the ER and/or Golgi apparatus 39, 40. Interestingly, like ZNT7, ZIP7 has also been indicated to interact with CD40 during a protein–protein interaction screening using affinity capture‐mass spectrometry 22. Although the physical interaction of ZIP7 with CD40 needs to be further confirmed by coimmunoprecipitation, our results clearly indicate that zinc is required for the initial stage of immune cell activation in response to extracellular stimuli. It is likely that ZNT7 and ZIP7 coordinate together to control cellular signal transductions in immune cells during early phases of cellular response to external stimuli by delivery of zinc to the local CD40 signalosome/trafficking components, or by increase in the cytoplasm zinc level for phosphatase inhibition.

In the present study, we observed that phosphorylation of IKKs was up‐regulated by *ZNT7*KD in Raji B cells in a CD154‐independent manner, which is consistent with previous reports demonstrating that up‐regulation of the NF‐κB signal transduction pathway is one of the signature molecular characteristics in most aggressive B lymphoma cells 16. It has been shown that constitutive NF‐κB activation in B lymphoma is required for lymphocyte proliferation and survival, and it is a key pathogenetic factor in lymphoma 16. In B lymphocytes, receptor self‐oligomerization, such as cluster of differentiation 30 (CD30) self‐oligomerization 41, can recruit TRAFs to the signalosome and constitutively activate IKKs leading to constitutive activation of NF‐κB via both the canonical (through degradation of IκB) and alternative (through partial proteolytic digestion of NF‐κB2/p100 to p52) signaling pathways to promote cell growth and survival 19. In addition, IKKs has been shown to directly phosphorylate other cellular proteins to promote cell growth. For example, IKKs can phosphorylate p53 and Foxo3a that are involved in tumor suppressor function 42, 43. In addition, IKKs are shown to control the mammalian target rapamycin complex 1 (mTORC1) activity by phosphorylation of TSC1 in the complex when AKTs are not activated 44. Activated mTORC1 is directly associated with cell survival by promoting oncogene translations 44, 45. Interestingly, in the present study, we also found that AKT phosphorylation levels were constitutively down‐regulated in *ZNT7*KD Raji B lymphocytes. We showed that CD154 stimulation did not dramatically increase the activity of AKTs in the B cell, which could favor the IKKs‐mTORC1‐oncoprotein translation pathway leading to cell survival and tumorigenesis. This result agrees with our previous findings demonstrating *ZNT7* KO or knockdown can negatively impact the insulin‐mediated AKT activation in muscle and fat tissues leading to down‐regulation of glucose uptake into myotubes and adipocytes 30, 31. Together, our study suggests that ZNT7 participates in cellular signaling pathways in regulation of growth and survival of B lymphocytes. *ZNT7*KD may promote B cell growth and/or survival leading to tumorigenesis. Indeed, we have previously shown that *ZNT7* KO in mice promotes prostate cancer development and metastasis via inhibition of apoptosis of prostate epithelial cells 46.

In conclusion, the present study demonstrates that ZNT7 physically interacts with CD40 and *ZNT7*KD has a negative impact on the expression of CD40 on the cell surface of B lymphocytes. As a result, *ZNT7*KD leads to down‐regulation of the CD154‐CD40‐mediated p38 MAPK signaling transduction, whereas OE of *ZNT7* up‐regulates p38 MAPK activity in Raji B lymphocytes. We also demonstrate that *ZNT7*KD in B lymphocytes constitutively up‐regulates IKK phosphorylation and down‐regulates AKT phosphorylation, which may be associated with B‐cell survival.

## Materials and methods

### Cell culture

Raji B lymphocytes were purchased from American Type Culture Collection (ATCC, Manassas, VA, USA). Raji B cells were grown as a suspension culture in RPMI 1640 complete media (ThermoFisher Scientific, Carlsbad, CA, USA), supplemented with 10% FBS, 5 mm GlutaMAX™, and 100 U·mL^−1^ penicillin 100 U·mL^−1^ streptomycin solution (ThermoFisher Scientific). Cells were maintained in this complete medium at a density between 5 × 10^4^ and 1 × 10^6^ cells·mL^−1^ in a humidity controlled incubator at 37 °C and 5% CO_2_.

### Plasmid constructions

To generate GST‐tagged mouse and human *ZNT7* expressing plasmids, the entire open reading frame (ORF) sequence of mouse *Znt7* or human *ZNT7* was PCR‐amplified, purified and subsequently inserted into pGEX‐4T3 (Promega Corp., Madison, WI, USA) in which *Znt7* (mouse) or *ZNT7* (human) was placed in‐frame downstream to the GST gene. To obtain a Myc‐tagged human CD40 full length or a Myc‐tagged C‐terminal end of the *CD40*, the entire ORF sequence of *CD40* and the cDNA sequence encoding the last 62 amino acid of *CD40* were PCR‐amplified using primers with a *Hin*dIII site incorporated immediately after the first methionine codon or the lysine_216_ codon of *CD40* and *Bam*HI site right before the stop codon of *CD40*. A methionine codon was also added into the forward primer for the PCR amplification of *CD40* C‐terminal cDNA fragment so that the cDNA could be translated in the bacteria. The *CD40* or *CD40C* ORF fragment was then inserted into a modified pGEX‐4T3 vector (pGEXBl) along with a MycHis‐tag, which was fused in‐frame at the C‐terminal end of *CD40* or *CD40C*. The pGEXBl was generated by removing the whole GST gene by *Hin*dIII‐*Bam*HI digestion of pGEX‐4T3. As such, the final *CD40‐* or *CD40C*‐expressing plasmid would only express CD40/MycHis or CD40C/MycHis fusion protein. To generate V5‐tagged human *ZNT7* expressing plasmids for mammalian cell expression, the human *ZNT7* ORF sequence was PCR‐amplified and inserted in‐frame along with a V5 cDNA sequence (ligated to the C‐terminal end) into the *Bam*HI and *Xho*I sites of pcDNA6 (Life Technology, Carlsbad, CA, USA). All plasmids generated for this study were validated by sequencing.

### Expression and purification of fusion proteins in bacteria

Plasmids, including pGEX/hZNT7, pGEX/mZnT7, pGEXBl/hCD40, and pGEXBl/hCD40C, were transformed into *Escherichia coli* strain BL21(DE3)pLysS. For ZNT7/ZnT7 expression, two bacterial colonies were inoculated into 3 mL LB containing 0.1 mg·mL^−1^ ampicillin and cultured overnight. Next day culture was diluted 1/50 and grown for 1.5 h. EDTA (1 mm) and IPTG (0.1 mm) was added into the culture and the cultures were incubated at 37 °C for another 1.5 h with shaking. For CD40 or CD40C expression, bacterial colonies were inoculated into 2× YTA containing 0.1 mg·mL^−1^ ampicillin and cultured overnight. The culture was then diluted 1/50 and incubated at 37 °C for another 1.5 h with shaking. IPTG (0.1 mm) was then added and the culture was further incubated at 37 °C for 2 h with shaking. After incubation, bacterial cells were pelleted, washed once with ice‐cold 1× PBS. The bacteria were then resuspended in GST binding/wash buffer (1/25 original culture volume; Promega) plus a tablet of complete mini protease inhibitor (Roche, South San Francisco, CA, USA) and 1 mg·mL^−1^ lysosome (Sigma‐Aldrich). The mixture was incubated at room temperature (RT) for 30 min. Four microliters of DNase (1 U·μL^−1^) and CaCl_2_ (0.5 mm) was then added and the mixture was further incubated for 30 min at RT. Bacteria were lysed by freeze/thaw cycles for eight times and sonicated with a Digital Sonifier® Cell Disruptor model 450 (Branson Ultrasonics, Danbury, CT, USA) at 18% for 10 s (0.5 s with 1 s break) three times in an ice water bath. The bacterial debris was removed by centrifugation.

The bacterial lysate (200 μL) containing GST/hZNT7 or GST/mZnT7 fusion protein was diluted in 250 μL GST‐binding buffer. MagneGST magnetic beads (100 μL, prewashed with GST‐binding buffer) were added to the lysate and the mixture was incubated at 4 °C overnight with rotating. The supernatant was then removed on a magnetic stand and the beads were washed and ready for protein–protein interaction assay (see below). CD40/Myc and CD40C/Myc fusion proteins were purified using HisPur™ Cobalt Purification Kit according to the manufacturer's instruction (ThermoFisher Scientific).

### Interaction of ZNT7 with CD40

Purified CD40/Myc or CD40C/Myc fusion protein was mixed with magnetic beads bound with either human ZNT7 or mouse ZnT7 and incubated at 4 °C for overnight in GST‐binding buffer with rotating. Unbounded protein was removed by centrifugation and the beads were washed with 400 μL of GST‐binding buffer five times. The CD40/ZnT7, CD40C/ZNT7, or CD40C/ZnT7 complex was then released from the beads in 2× protein loading buffer (BioRad, Hercules, CA, USA) at 99 °C for 10 min followed by western blot analysis.

### Generation of stable *ZNT7* knockdown and *ZNT7*‐overexpressing Raji cell lines

A stable *ZNT7*KD line was established by transduction of Raji cells with lentiviruses expressing shRNA complementary to the human *SLC30A7* (*ZNT7*) mRNA (Sigma‐Aldrich). Raji cells (5 × 10^4^) seeded in a 12‐well cell culture plate were incubated with 8 μg·mL^−1^ polybrene and 1.5 lentiviral particles/cell in RPMI 1640 media for 24 h. After incubation, viral particles were removed and the cells were incubated with RPMI 1640 for another 24 h at 37 °C. The stable cells were selected by incubating cells in the medium containing 1 μg·mL^−1^ puromycin for 10 days. After selection, the cells were pooled and used for subsequent experiments.

The *ZNT7*‐overexpressing Raji cell line was generated by transfecting cells with pcDNA6 (Life Technologies, Carlsbad, CA, USA) or pcDNA6/*ZNT7* plasmid by electroporation, according to the manufacturer's protocol (Cell‐Porator, Life Technologies). After 48 h of incubation at 37 °C, dead cells were removed by seeding the cells in 10 mL of fresh RPMI without FBS in T‐25 flasks for 5 h. Live cells were then incubated in the complete medium containing 10 μg·mL^−1^ml blasticidin. After a 7‐day selection period, single cell lines were selected in the complete medium containing 5 μg·mL^−1^ blasticidin by limiting dilution 47. Three individual *ZNT7*‐overexpressing cell clones that expressed ~ 50% more *ZNT7* mRNA than the controls were selected for the subsequent experiments. The control Raji cells (2 lines) were the pools of blasticidin resistance cells.

### Antibodies

Antibodies against p38 MAPK, phosphor‐p38 MAPK (Thr180/Tyr182), AKT, phosphor‐AKT (Ser472), phosphor‐IKKα (Ser176)/IKKβ (Ser177), and phosphor‐JNK (Thr183/Try185) were purchased from Cell Signaling Technology (Danvers, MA, USA). Antibodies against human CD40 and c‐Myc were purchased from Santa Cruz Biotechnology (Santa Cruz, CA, USA). Antibodies against binding immunoglobulin protein (BIP) and human ZNT7 were obtained from Eurogentec (Fremont, CA, USA) and Proteintech (Rosemont, IL, USA), respectively. The antibody against mouse ZnT7 was described previously 23. A biotin‐conjugated antibody against human CD40 (clone EA‐5) was purchased from Life Span BioSciences, Inc. (Seattle, WA, USA). ACTB and tubulin antibodies were purchased from Sigma‐Aldrich. Horseradish peroxide‐conjugated secondary antibodies were obtained from Cell Signaling Technology.

### Western blot analysis

For TPEN (*N*,*N*,*N*'*N*'‐Tetrakis(2‐pyridylmethyl)ethylenediamine; Santa Cruz Biotechnology) treatment experiments, Raji cells were seeded into six‐well plates at 3 × 10^6^ per well in serum‐free RPMI 1640 media with either TPEN concentrations at 0, 2.5, 5, 7.5, or 10 μm, or with the vehicle (ethanol) at 0.01% (v/v) at 37 °C for 2 h. Where indicated, ZnSO_4_ was also added into the well at 5 μm. Cells were then washed and stimulated with either 0 or 100 ng·mL^−1^ CD154 (Cell Signaling Technologies) at 37 °C for 10 min. The cells were then washed with ice‐cold 1× PBS, lysed in M‐PER™ buffer (0.15 mL) containing 1× Halt™ Protease and Phosphatase Inhibitor Cocktail (ThermoFisher Scientific) and stored at −80 °C until use. For experiments examining the activation of signaling pathways after CD154 stimulation in zinc repletion condition, Raji cells (3 × 10^6^ per well) were seeded into six‐well plates in serum‐free RPMI 1640 media with or without ZnSO_4_ (5 μm) and incubated at 37 °C for 2 h. After incubation, cells were stimulated with 100 ng·mL^−1^ CD154 for 0, 10, or 20 min. Cells were lysed and stored after stimulation in M‐PER™ buffer as described above.

For detection of endogenous CD40 and ZNT7 proteins, Raji cells were cultured in the complete RPMI media for 48 h and 3 × 10^6^ cells were lysed. For determination of kinase activities, Raji cells including *ZNT7*KD and *ZNT7*‐overexpressing lines, cells were seeded into six‐well plates at 3 × 10^6^ per well in serum‐free RPMI 1640 media and incubated at 37 °C for 2 h. Cells were then stimulated with CD154 (100 ng·mL^−1^) at 37 °C for 0, 5, 10, or 20 min. After stimulation, cells were lysed as described above. For experiments examining the surface expression of CD40, cells were seeded and stimulated for the indicated time points as described above. Cells were then washed with ice‐cold 1× PBS and fixed with freshly made 4% paraformaldehyde at RT for 10 min on ice. Cells were incubated with 5% BSA for 30 min at RT followed by a CD40 antibody (1.25 μg·mL^−1^; LifeSpan BioSciences) at RT for 1 h. Cells were washed with 1× PBS and lysed as described above.

Lysate was sonicated for 10 s, with 0.5 s on and 1.0 s off and at an amplitude of 10% on a Digital Sonifier^®^ Cell Disruptor model 450 (Branson Ultrasonics). Protein concentration was determined by a bicinchoninic acid kit (BioRad) prior to loading on 10% Criterion™ TGX™ precast gels (BioRad). Western blot assays were performed as described previously 31. Protein bands were visualized using a chemiluminescent substrate (either a Clarity™ Western ECL substrate (BioRad) or a SuperSignal^®^ West Femto Maximum Sensitivity Substrate; ThermoFisher Scientific) on a ChemiDoc XRS+ System (BioRad) and quantitated using image Lab software (BioRad).

### Immunofluorescent microscopy

Raji B lymphocytes were seeded in 96‐well plates (black/clear) or slide chambers at 0.75 × 10^5^ per well and incubated in serum‐free RPMI 1640 media at 37 °C for 4 h. Cells were fixed with 4% paraformaldehyde. Cells that were used for ZNT7 detection were permeabilized with 0.4% saponin (Sigma‐Aldrich). Anti‐CD40 and anti‐ZnT7 primary antibodies were applied, followed by Alexa 488‐conjugated goat anti‐rabbit antibodies. Photomicrographs were obtained by either a Zeiss Axiovert 40 CFL (Zeiss International, Oberkochen, Germany) or a Nikon Eclipse 800 microscope (Nikon Instruments Inc., Melville, NY, USA).

### Immunoprecipitation

Raji B cells (3 × 10^6^) were treated with 100 ng·mL^−1^ CD154 (Cell Signaling Technology) or vehicle for 10 min. Cells were washed with 1× ice‐cold PBS and cross‐linked with 1% paraformaldehyde in 1× PBS for 7 min. Cells were pelleted and resuspended in 1 mL 1.25 m glycine/PBS to stop the cross‐linking reaction. Cells were washed with 1.25 m glycine twice and lysed in 500 μL M‐PER™ containing 1× Halt™ Protease and Phosphatase Inhibitor Cocktail (ThermoFisher Scientific). Protein concentration was determined by a bicinchoninic acid assay (BioRad). Lysate (250 μg) was diluted 1 : 1 in 1× PBS and incubated with 1 μg normal rabbit IgG (Santa Cruz Biotechnology) at RT for 30 min. Goat anti‐(rabbit IgG) magnetic beads (200 μg, New England BioLabs, Ipswich, MA, USA) were added and incubated at RT for another 30 min. The precleared lysate was then incubated with a ZNT7 antibody (1 μg) at RT for 1 h. Prewashed magnetic beads (200 μg) were then added and incubated overnight at 4 °C. The beads were then washed with 1 mL diluted M‐PER (1 : 1 with 1× PBS). The protein complex was released from the beads in 2× protein loading buffer (BioRad) at 99 °C for 10 min followed by western blot analysis.

### Total RNA isolation, cDNA synthesis, and quantitative PCR

*ZNT7* knockdown, ZNT7‐overexpressing, and control Raji cells were seeded into 12‐well plates in duplicate at 1 × 10^6^ per well in RPMI 1640 complete media. After 2 h of incubation at 37 °C, the cells were treated with CD154 (100 ng·mL^−1^) or mock treated. The cells were further incubated for 0, 2, 4, or 6 h at 37 °C. The cells were then harvested at the above indicated time points for total RNA purification using a TRIzol^®^ reagent (ThermoFisher Scientific) according to the manufacturer's protocol. Total RNA (1 μg) was converted to cDNA using an iScript™ Reverse Transcription Supermix for RT‐qPCR (BioRad) following manufacturer's instructions. Quantitative PCR reactions were performed in an ABI PRlSM^®^ 7900 HT Sequence Detection System (Applied Biosystems, Foster City, CA, USA) using an iTaq™ universal SYBR^®^ Green supermix (BioRad). Fold difference of the target gene expression was calculated using a ▵Ct method as described previously 48. The expression of either *ACTB* or *TBP* was used as a reference for quantitation of the expression of the target gene. Primer pairs used in this study are listed in Table 1.

**Table 1:** Primer sequences

Gene	Forward primer (5′→3′)	Reverse primer (5′→3′)
ZNT7	GCAGATCCTATCTGTTCAATTCT	TTCTAATAGGGGAGGAGTTCT
ACTB	ACTGGCATCGTGATGGACTC	AGGTAGTCAGTCAGGTC
TBP	ACAGTGAATCTTGGTTGTAAAC	GCAGCAAACCGCTTGGGATTA

### Statistical analysis

Significant differences between two test groups were determined using an unpaired Student's *t* test. Differences were considered significant at *P *<* *0.05. Data were presented as either mean ± SE or mean ± SD.

## Author contributions

LH planned and designed experiments; ST and CPK performed protein–protein interaction experiments using purified fusion proteins from bacterial lysate. ST and PO performed signaling transduction assays. PO performed quantitative RT‐PCR, immunocytochemistry, and immunoprecipitation using protein lysate from Raji B lymphocytes; CPK and YC generated all plasmids for the study. CPK also assisted western blot assays and immunocytochemistry. LH analyzed data; LH and ST wrote the draft manuscript. PO, CPK, and YC edited the manuscript. All authors have read and approved the final manuscript.
